# Prediction of response to remission induction therapy by gene expression profiling of peripheral blood in Japanese patients with microscopic polyangiitis

**DOI:** 10.1186/s13075-017-1328-7

**Published:** 2017-05-31

**Authors:** Akihiro Ishizu, Utano Tomaru, Sakiko Masuda, Ken-ei Sada, Koichi Amano, Masayoshi Harigai, Yasushi Kawaguchi, Yoshihiro Arimura, Kunihiro Yamagata, Shoichi Ozaki, Hiroaki Dobashi, Sakae Homma, Yasunori Okada, Hitoshi Sugiyama, Joichi Usui, Naotake Tsuboi, Seiichi Matsuo, Hirofumi Makino, for the Research Committee of the Intractable Vasculitis Syndrome and the Research Committee of the Intractable Renal Disease of the Ministry of Health, Labour, and Welfare of Japan

**Affiliations:** 1 Faculty of Health Sciences, Hokkaido University, Kita-12, Nishi-5, Kita-ku, Sapporo, 060-0812 Japan; 2 Department of Pathology, Hokkaido University Graduate School of Medicine, Sapporo, Japan; 3 Department of Nephrology, Rheumatology, Endocrinology and Metabolism, Okayama University Graduate School of Medicine, Dentistry and Pharmaceutical Sciences, Okayama, Japan; 4 Department of Rheumatology and Clinical Immunology, Saitama Medical Center, Saitama Medical University, Saitama, Japan; 5 Department of Pharmacovigilance, Graduate School of Medical and Dental Sciences, Tokyo Medical and Dental University, Tokyo, Japan; 6 Institute of Rheumatology, Tokyo Women’s Medical University, Tokyo, Japan; 7 Nephrology and Rheumatology, First Department of Internal Medicine, Kyorin University School of Medicine, Tokyo, Japan; 8 Department of Nephrology, Faculty of Medicine, University of Tsukuba, Ibaraki, Japan; 9 Division of Rheumatology and Allergology, Department of Internal Medicine, St. Marianna University School of Medicine, Kawasaki, Japan; 10 Division of Hematology, Rheumatology and Respiratory Medicine, Department of Internal Medicine, Faculty of Medicine, Kagawa University, Kagawa, Japan; 11 Department of Respiratory Medicine, Toho University Omori Medical Center, Tokyo, Japan; 12 Department of Pathology, Keio University School of Medicine, Tokyo, Japan; 13 Department of Human Resource Development of Dialysis Therapy for Kidney Disease, Okayama University Graduate School of Medicine, Dentistry and Pharmaceutical Sciences, Okayama, Japan; 14 Department of Nephrology, Internal Medicine, Nagoya University Graduate School of Medicine, Nagoya, Japan; 15 Okayama University Hospital, Okayama, Japan

**Keywords:** Microscopic polyangiitis, Prediction of response to treatment, Gene profiling, Peripheral blood

## Abstract

**Background:**

Microscopic polyangiitis (MPA), which is classified as an anti-neutrophil cytoplasmic antibody (ANCA)-associated small vessel vasculitis, is one of the most frequent primary vasculitides in Japan. We earlier nominated 16 genes (*IRF7*, *IFIT1*, *IFIT5*, *OASL*, *CLC*, *GBP-1*, *PSMB9*, *HERC5*, *CCR1*, *CD36*, *MS4A4A*, *BIRC4BP*, *PLSCR1*, *DEFA1/DEFA3*, *DEFA4*, and *COL9A2*) as predictors of response to remission induction therapy against MPA. The aim of this study is to determine the accuracy of prediction using these 16 predictors.

**Methods:**

Thirty-nine MPA patients were selected randomly and retrospectively from the Japanese nationwide RemIT-JAV-RPGN cohort and enrolled in this study. Remission induction therapy was conducted according to the Guidelines of Treatment for ANCA-Associated Vasculitis published by the Ministry of Health, Labour, and Welfare of Japan. Response to remission induction therapy was predicted by profiling the altered expressions of the 16 predictors between the period before and 1 week after the beginning of treatment. Remission is defined as the absence of clinical manifestations of active vasculitis (Birmingham Vasculitis Activity Score 2003: 0 or 1 point). Persistent remission for 18 months is regarded as a “good response,” whereas no remission or relapse after remission is regarded as a “poor response.”

**Results:**

“Poor” and “good” responses were predicted in 7 and 32 patients, respectively. Five out of 7 patients with “poor” prediction and 1 out of 32 patients with “good” prediction experienced relapse after remission. One out of 7 patients with “poor” prediction was not conducted to remission. Accordingly, the sensitivity and specificity to predict poor response was 85.7% (6/7) and 96.9% (31/32), respectively.

**Conclusions:**

Response to remission induction therapy can be predicted by monitoring the altered expressions of the 16 predictors in the peripheral blood at an early point of treatment in MPA patients.

## Background

Microscopic polyangiitis (MPA), which is classified as an anti-neutrophil cytoplasmic antibody (ANCA)-associated small vessel vasculitis [1], is one of the most frequent primary vasculitides in East Asia. In Japan, Ozaki et al. conducted a prospective clinical trial on newly diagnosed patients with MPA in which they were administered the remission induction therapy according to disease severity [2]. Briefly, the MPA patients were stratified into three categories based on disease severity, namely mild form, severe form, and most severe form. The mild form included patients with slight disorder of one or more organs, a renal-limited type (except rapidly progressive glomerulonephritis (RPGN)), and a pulmonary-limited type (except pulmonary hemorrhage). The severe form consisted of patients with a generalized type (MPA with involvement of more than two organs), pulmo-renal type (glomerulonephritis plus either limited pulmonary hemorrhage or extended interstitial pneumonia), and RPGN. The most severe form included patients with diffuse alveolar hemorrhage, intestinal perforation, acute pancreatitis, cerebral hemorrhage, or concurrent presence of anti-glomerular basement membrane antibodies. After the establishment of diagnosis, the patients were treated according to the following protocols. 1) The mild form: low-dose corticosteroids (0.3–0.6 mg/kg/day) were administered; oral immunosuppressive agents (cyclophosphamide or azathioprine, 0.5–1.0 mg/kg/day or 25–75 mg/day, respectively) were optional. 2) The severe form: high-dose corticosteroids (0.6–1.0 mg/kg/day) and oral cyclophosphamide (0.5–2.0 mg/kg/day) were given; intravenous methylprednisolone (0.5–1.0 g/day for 3 days) was considered as an alternative. Instead of oral administration, the use of intravenous cyclophosphamide (0.5–0.75 g/m^2^ monthly) was also allowed. 3) Most severe form: plasmapheresis (2.0–3.0 L/day for 3 days) was employed together with the regimen for the severe form described above.

After 18 months, the outcome of 47 patients, comprising of 22 with mild disease, 23 with severe disease, and 2 with the most severe disease, were analyzed. Remission, which is defined as the absence of clinical manifestations of active vasculitis (Birmingham Vasculitis Activity Score 2003: 0 or 1 point), was achieved in 42 out of 47 patients (remission rate, 89.4%). Among the 42 patients, 8 showed relapse of the disease, which is defined as the recurrence of at least one manifestation of vasculitis (recurrence rate, 19.0%). Ultimately, 5 out of 47 patients died (mortality rate, 10.6%).

These results demonstrate that the suggested therapeutic protocols are applicable for patients with MPA, but the possibility of relapse is indicated and, in the worst case scenario, death may occur regardless of the treatment. We have considered that, if response to the remission induction therapy would be predicted at an early point during the therapy, meticulous follow-up or application of additional regimens to the treatment could expectedly improve the outcome. For this purpose, we focused on the typical alteration of gene expressions after treatment in “good responders” who were conducted to persistent remission. We considered the prediction of “poor responders” who would not be conducted to remission or would relapse after remission when such typical alteration of gene expressions was not observed in the peripheral blood. Based on the results obtained through the transcriptome analysis, we nominated 16 genes from the comprehensive 8793 genes as predictors of response to remission induction therapy in MPA [3]. The 16 predictors included *interferon (IFN) regulatory factor (IRF)7*, *IFN-induced protein with tetratricopeptide repeats (IFIT)1*, *IFIT5*, *2′-5′-oligoadenylate synthetase-like (OASL)*, *Charcot-Leyden crystal protein (CLC)*, *guanylate binding protein 1 (GBP-1)*, *proteasome (prosome macropain) subunit*, *beta type*, *9 (PSMB9)*, *hect domain and RLD (HERC)5*, *chemokine (C-C motif) receptor 1 (CCR1)*, *CD36*, *membrane-spanning 4-domains*, *subfamily A*, *member 4 (MS4A4A)*, *XIAP-associated factor-1 (BIRC4BP)*, *phospholipid scramblase 1 (PLSCR1)*, *defensin α1 and α3 (DEFA1/DEFA3)*, *defensin α4 (DEFA4)*, and *collagen type IX α2 (COL9A2)*.

In the present study, we determined the accuracy of prediction with the use of the aforementioned 16 predictors on 39 MPA patients who were selected randomly and retrospectively from the Japanese nationwide RemIT-JAV-RPGN cohort [4].

## Methods

### Patient cohorts

Currently in Japan, there are two nationwide cohorts of vasculitides, namely the JMAAV cohort [2] and RemIT-JAV-RPGN cohort [4]. In the JMAAV cohort, diagnosis of MPA was made according to the diagnostic criteria for MPA of the Research Group of Intractable Vasculitis, the Ministry of Health, Labour, and Welfare (MHLW) of Japan [5], whereas the diagnostic algorithm for primary systemic vasculitis proposed by the European Medicines Agency (EMEA) [6] was adopted in the RemIT-JAV cohort. Although some patients diagnosed as MPA according to the Japanese MHLW criteria were categorized into granulomatosis with polyangiitis (GPA) in the EMEA algorithm, most patients categorized into MPA in the EMEA algorithm were also diagnosed as MPA according to the Japanese MHLW criteria [7]. Therefore, it is conceivable that MPA patients in the RemIT-JAV-RPGN cohort appear to manifest a typical phenotype of MPA. In both cohorts, only patients without any prior treatment for MPA were enrolled. Patients in the RemIT-JAV-RPGN cohort received remission induction therapy according to the Japanese MHLW Guidelines of Treatment for ANCA-Associated Vasculitis that was reflected in the JMAAV protocol. Thirty-nine MPA patients were selected randomly and retrospectively from the RemIT-JAV-RPGN cohort and included in this study. The baseline characteristics of these MPA patients and the given remission induction therapy are shown in Table 1. Written informed consent was obtained from all the patients at their respective institutes where they were being treated. The study protocol was approved by the respective institutional ethics committees.

**Table 1:**
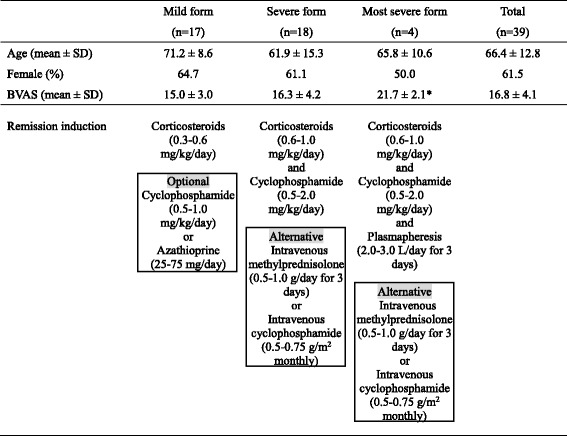
Baseline characteristics of MPA patients and given remission induction therapy

*p < 0.05 vs mild form

*BVAS* Birmingham Vasculitis Activity Score, *MPA* microscopic polyangiitis, *SD* standard deviation

### Blood samples and gene expression profiling

Peripheral blood samples (10 mL) were obtained before and 1 week after the beginning of remission induction therapy. Total RNAs were extracted using the PAXgene Blood RNA System (BD, Franklin Lakes, NJ, USA). High throughput real-time reverse transcription polymerase chain reaction (PCR; Applied Biosystems, Carlsbad, CA, USA) was applied to quantify the expression of the 16 genes (*IRF7*, *IFIT1*, *IFIT5*, *OASL*, *CLC*,* GBP-1*, *PSMB9*, *HERC5*, *CCR1*, *CD36*, *MS4A4A*, *BIRC4BP*, *PLSCR1*, *DEFA1/DEFA3*, *DEFA4*, and *COL9A2*) which were identified as predictors in the earlier study [3].

### Regression formula for the prediction index that represents response to remission induction therapy

Prior to this study, we have determined the regression formula that reflected the altered expression of the 16 genes by remission induction therapy and the response to the therapy by employing 22 MPA patients from the JMAAV cohort. For this purpose, the Ct value of real-time PCR was applied. The Ct value represents the cycle number in which the PCR products reach the threshold level. The expression level of the target gene was shown as ΔCt (ΔCt = Ct value of the target gene – Ct value of the housekeeping *β-actin* gene). Next, the changed amount of expression of the target gene by the treatment was shown as ΔΔCt (ΔΔCt = ΔCt 1 week after the beginning of treatment – ΔCt before treatment). It is considered that when ΔΔCt is 1, the expression level of the target gene before treatment is twofold higher than 1 week after the beginning of treatment. Accordingly, when the expression level of the target gene before treatment is set as 1, the fold-expression of the target gene 1 week after the beginning of treatment is shown as 2^–ΔΔCt^. Subsequently, the response to the treatment was replaced by a dummy number, wherein “good response (persistent remission)” was regarded as 0 and “poor response (relapse after remission or no remission)” was regarded as 1. After these preparations, multiple regression analysis was conducted concerning the 22 MPA patients, including 17 good responders and 5 poor responders (4 patients relapsed after remission and remission was not achieved in 1 patient).

### Prediction of response to remission induction therapy

In this analysis, response to the remission induction therapy was replaced by dummy numbers, 0 and 1, wherein 0 means “good response” and 1 means “poor response.” For the next discrimination analysis, we plotted the receiver operating characteristic (ROC) curve. However, the ROC curve was not suitable for this case (data not shown). Thus, we determined the boundary value as the mean value of the expected prediction indices of the 22 patients. Since 0 was applied to 17 patients and 1 was applied to 5 patients, the mean value of the total of 22 patients was 0.23. Therefore, the prediction index of less than 0.23 predicts “good response,” whereas the value greater than 0.23 predicts “poor response.”

### Discrimination analysis

The accuracy of prediction was evaluated by employing another 39 MPA patients who were selected randomly and retrospectively from the RemIT-JAV-RPGN cohort. These patients were completely different from those enrolled in the derivation of the regression formula for the prediction index.

## Results

### Determination of regression formula for the prediction index that represents response to remission induction therapy

In our earlier study, we conducted the comprehensive transcriptome analysis using peripheral blood samples obtained before and 1 week after the beginning of remission induction therapy on 12 MPA patients selected randomly from the JMAAV cohort (Cohort 1) [3]. Results demonstrated that the expressions of 88 genes were significantly altered after the treatment in 9 “good responders.” This characteristic alteration of gene expression was not observed in 3 “poor responders.” We selected 30 genes that showed the statistically top values among the 88 genes.

Next, in order to identify the most valuable genes for prediction of response to the treatment, the logistic regression analysis with stepwise method was carried out on the 30 genes using the add-in Excel software 2012. For this purpose, we employed another cohort, Cohort 2, selected randomly from the JMAAV patients. In brief, 16 genes were selected randomly from the 30 genes at first, and then the influence of the genes on the prediction was calculated. Thereafter, the gene which showed the minimum influence on the prediction was replaced by another gene among the remaining 14 genes. This operation was repeated until all genes were used. Subsequently, the gene with the minimum influence on the prediction was excluded one by one until the last gene remained. All combinations of genes were examined for prediction of the response to the treatment. Ultimately, the 16 genes, including *IRF7*, *IFIT1*, *IFIT5*, *OASL*, *CLC*, *GBP-1*, *PSMB9*, *HERC5*, *CCR1*, *CD36*, *MS4A4A*, *BIRC4BP*, *PLSCR1*, *DEFA1/DEFA3*, *DEFA4*, and *COL9A2*, were nominated as the most valuable genes for prediction and, at the same time, the regression formula for prediction of response to remission induction therapy was determined as follows. The contribution of the 16 genes to prediction is shown in Table 2.

**Table 2:** Contribution of 16 genes to prediction

	Coefficient	Standard error	*P* value
Intercept	0.84	0.16	0.004
*IRF7*	0.74	0.17	0.008
*IFIT1*	–0.32	0.09	0.015
*IFIT5*	–1.43	0.21	0.001
*OASL*	–0.18	0.16	0.301
*CLC*	0.05	0.02	0.037
*GBP1*	–0.84	0.20	0.009
*PSMB9*	0.38	0.35	0.321
*HERC5*	0.37	0.10	0.013
*CCR1*	–0.91	0.26	0.012
*CD36*	–0.84	0.26	0.024
*MS4A4A*	0.43	0.21	0.099
*BIRC4BP*	0.93	0.09	0.000
*PLSCR1*	0.72	0.27	0.047
*DEFA/DEFA3*	0.01	0.01	0.367
*DEFA4*	–0.02	0.02	0.311
*COL9A2*	–0.06	0.02	0.015

Prediction index = 0.84 + (0.74) × (2^–ΔΔCt^ of *IRF7*) + (–0.32) × (2^–ΔΔCt^ of *IFIT1*) + (–1.44) × (2^–ΔΔCt^ of *IFIT5*) + (–0.18) × (2^–ΔΔCt^ of *OASL*) + (0.05) × (2^–ΔΔCt^ of *CLC*) + (–0.84) × (2^–ΔΔCt^ of *GBP1*) + (0.38) × (2^–ΔΔCt^ of *PSMB9*) + (0.37) × (2^–ΔΔCt^ of *HERC5*) + (–0.91) × (2^–ΔΔCt^ of *CCR1*) + (–0.84) × (2^–ΔΔCt^ of *CD36*) + (0.43) × (2^–ΔΔCt^ of *MS4A4A*) + (0.93) × (2^–ΔΔCt^ of *BIRC4BP*) + (0.72) × (2^–ΔΔCt^ of *PLSCR1*) + (0.01) × (2^–ΔΔCt^ of *DEFA1/DEFA3*) + (–0.02) × (2^–ΔΔCt^ of *DEFA4*) + (–0.06) × (2^–ΔΔCt^ of *COL9A2*).

### Relevance of regression formula and boundary value for the prediction of response to remission induction therapy

**Fig. 1:**
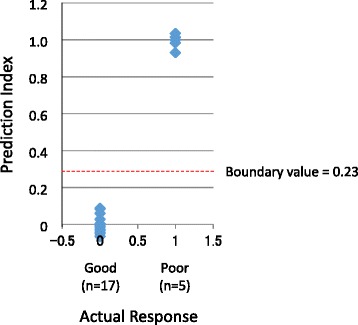
Relevance of regression formula and boundary value for prediction of response to remission induction therapy. The prediction indices and actual responses of the 22 training patients, including 17 “good responders” and 5 “poor responders,” were plotted together with the boundary value. The boundary value was determined as the mean value of expected prediction indices of the 22 patients. Since 0 was applied to 17 patients and 1 was applied to 5 patients, the mean value of the total of 22 patients was 0.23. Therefore, the prediction index of less than 0.23 predicts “good response,” whereas the value greater than 0.23 predicts “poor response”

In order to determine the relevance of the regression formula and the boundary value for the prediction of response to remission induction therapy, we plotted the prediction indices and actual responses of the 22 training samples together with the boundary value (Fig. 1). Results demonstrate the relevance of the regression formula and the boundary value for prediction.

### Accuracy of prediction of response to remission induction therapy

**Fig. 2:**
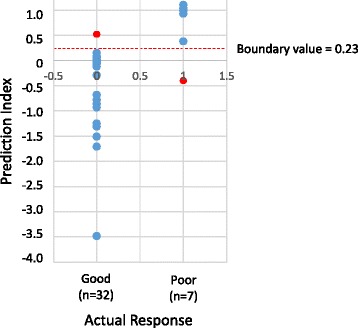
Prediction indices of 39 MPA patients. The prediction indices and actual responses of the 39 MPA patients, including 32 “good responders” and 7 “poor responders,” were plotted together with the boundary value. *Red dots* represent patients whose prediction is inconsistent with actual response

To evaluate the accuracy of prediction, the correlation between prediction indices and actual responses to the remission induction therapy was analyzed concerning the next 39 MPA patients (Fig. 2). As a result, “poor” and “good” responses were predicted in 7 and 32 patients, respectively (Table 3). Five out of 7 patients with “poor” prediction and 1 out of 32 patients with “good” prediction experienced relapse after remission. One out of 7 patients with “poor” prediction was not conducted to remission. Accordingly, the sensitivity and specificity to predict poor response was 85.7% (6/7) and 96.9% (31/32), respectively. We determined the 95% confidence intervals of the sensitivity and specificity as 0.421–0.996 and 0.838–0.999, respectively.

**Table 3:** Predicted and actual responses to remission induction therapy against microscopic polyangiitis (*n* = 39)

Prediction	Actual response	
	Poor	Good
Poor	6	1
Good	1	31

Table 4 demonstrates the detailed results with regard to disease severity. The overall accuracy of prediction is 94.9%, while the accuracy tends to decline as the disease severity increases.

**Table 4:** Accuracy of prediction

	Mild form		Severe form		Most severe form		Total	
	(*n* = 17)		(*n* = 18)		(*n* = 4)		(*n* = 39)	
Actual response	Poor	Good	Poor	Good	Poor	Good	Poor	Good
Prediction								
Poor	3	0	2	1	1	0	6	1
Good	0	14	0	15	1	2	1	31
Accuracy (%)	100		94.4		75.0		94.9	

## Discussion

Lyons and colleagues reported that the transcriptome analysis of leukocyte subsets before treatment enabled the identification of gene signatures of ANCA-associated vasculitis, including MPA [8]. Similarly, McKinney et al. reported that the transcription signature of CD8^+^ T cells before treatment could predict prognosis in autoimmune diseases, including MPA [9]. On the contrary, we have focused on the altered gene expression in the peripheral blood of MPA patients between the period before and 1 week after the beginning of remission induction therapy. Therefore, it would seem likely that the prediction was based on the effects of the therapeutic reagents included in the remission induction regimen.

Among the 16 genes used as predictors in this study, the expressions of *IRF7*, *IFIT1*, *IFIT5*, *OASL*, *CLC*, *GBP-1*, *PSMB9*, *HERC5*, *CCR1*, *CD36*, *MS4A4A*, *BIRC4BP*, and *PLSCR1* were decreased, whereas those of *DEFA1/DEFA3*, *DEFA4*, and *COL9A2* were increased after treatment in “good responders” [3]. The relation between the decrease in several IFN-related genes, such as *IRF7* [10], *IFIT1* [11], *IFIT5* [12], *OASL* [13], and *GBP-1* [14], after the anti-inflammatory immunosuppressive treatment and the “good response” could be profound. ANCA-associated vasculitis, including MPA, has not been regarded as a type 1 IFN-driven disease [8]. We consider the expressions of the IFN-related genes as very sensitive to immunosuppressive treatment. Thus, we assume that the alteration can be a good marker of the therapeutic effects, though the expressions are not signatures of the disease.

In addition, we noted that the decrease in *CLC* gene expression after treatment reflected the “good response.” CLC proteins are mainly expressed in eosinophils [15], and the eosinophil count is rapidly reduced by corticosteroid treatment; therefore, the reduction of eosinophils in the peripheral blood after treatment possibly predicts “good response.” However, we could not assess the relation between prediction and alteration of leukocyte counts or rates of subpopulations in the peripheral blood after the treatment. This is the limitation of this study. We hope to challenge the clarification of this issue in our future studies.

We have demonstrated that the response to remission induction therapy can be predicted by monitoring the altered expressions of the 16 predictors in the peripheral blood at an early point of treatment in MPA patients. On the other hand, the accuracy tends to decline according to the increase in disease severity, though there is no statistical power. A significantly bigger sample size can elucidate the benefits and disadvantages of the prediction in future studies.

Although improvement to increase the sensitivity to predict “poor responders” is needed, such prediction at an early point of treatment, if any, should be significant. This prediction can help us consider meticulous follow-up or application of additional regimens to the treatment for the patients who are predicted as “poor responders.”

## Conclusions

The response to remission induction therapy can be predicted by monitoring the altered expressions of the 16 predictors in the peripheral blood at an early point of treatment in MPA patients.

## Abbreviations

ANCA: Anti-neutrophil cytoplasmic antibody
BIRC4BP: XIAP-associated factor-1
CCR1: Chemokine (C-C motif) receptor 1
CLC: Charcot-Leyden crystal protein
COL9A2: Collagen type IX α2
DEFA1/DEFA3/DEFA4: Defensin α1, α3, and α4
EMEA: European Medicines Agency
GBP-1: Guanylate binding protein 1
HERC5: Hect domain and RLD
IFIT: Interferon-induced protein with tetratricopeptide repeats
IFN: Interferon
IRF: Interferon regulatory factor
MHLW: Ministry of Health, Labour, and Welfare
MPA: Microscopic polyangiitis
MS4A4A: Membrane-spanning 4-domains, subfamily A, member 4
OASL: 2′-5′-Oligoadenylate synthetase-like
PCR: Polymerase chain reaction PLSCR1, Phospholipid scramblase 1
PSMB9: Proteasome (prosome macropain) subunit, beta type, 9
ROC: Receiver operating characteristic
RPGN: Rapidly progressive glomerulonephritis
